# Evaluating the accuracy and relevance of ChatGPT responses to frequently asked questions regarding total knee replacement

**DOI:** 10.1186/s43019-024-00218-5

**Published:** 2024-04-02

**Authors:** Siyuan Zhang, Zi Qiang Glen Liau, Kian Loong Melvin Tan, Wei Liang Chua

**Affiliations:** Department of Orthopaedic Surgery, National University Health System, Level 11, NUHS Tower Block, 1E Kent Ridge Road, Singapore, 119228 Singapore

**Keywords:** ChatGPT, Artificial intelligence, Chatbot, Large language model, Total knee replacement, Total knee arthroplasty

## Abstract

**Background:**

Chat Generative Pretrained Transformer (ChatGPT), a generative artificial intelligence chatbot, may have broad applications in healthcare delivery and patient education due to its ability to provide human-like responses to a wide range of patient queries. However, there is limited evidence regarding its ability to provide reliable and useful information on orthopaedic procedures. This study seeks to evaluate the accuracy and relevance of responses provided by ChatGPT to frequently asked questions (FAQs) regarding total knee replacement (TKR).

**Methods:**

A list of 50 clinically-relevant FAQs regarding TKR was collated. Each question was individually entered as a prompt to ChatGPT (version 3.5), and the first response generated was recorded. Responses were then reviewed by two independent orthopaedic surgeons and graded on a Likert scale for their factual accuracy and relevance. These responses were then classified into accurate versus inaccurate and relevant versus irrelevant responses using preset thresholds on the Likert scale.

**Results:**

Most responses were accurate, while all responses were relevant. Of the 50 FAQs, 44/50 (88%) of ChatGPT responses were classified as accurate, achieving a mean Likert grade of 4.6/5 for factual accuracy. On the other hand, 50/50 (100%) of responses were classified as relevant, achieving a mean Likert grade of 4.9/5 for relevance.

**Conclusion:**

ChatGPT performed well in providing accurate and relevant responses to FAQs regarding TKR, demonstrating great potential as a tool for patient education. However, it is not infallible and can occasionally provide inaccurate medical information. Patients and clinicians intending to utilize this technology should be mindful of its limitations and ensure adequate supervision and verification of information provided.

**Supplementary Information:**

The online version contains supplementary material available at 10.1186/s43019-024-00218-5.

## Introduction

Total knee replacement (TKR) is one of the most common elective orthopaedic procedures performed today [1], helping countless patients with knee arthritis achieve improvements in pain, function and quality of life [2].

As the demand for and volume of TKRs rise, an increasing number of patients are turning to the internet for information regarding this procedure [3, 4]. Prior research has shown that up to two-thirds of patients considering elective orthopaedic procedures have used the internet as a source of information [4, 5]. This has coincided with the rising prominence of artificial intelligence (AI) chatbots such as Chat Generative Pretrained Transformer (ChatGPT) in recent years. Since its release in November 2022, ChatGPT has garnered great interest due to its ability to generate coherent and human-like responses across a wide range of topics – surpassing 100 million monthly active users in just 2 months and setting the record for the fastest growing application in history [6–8]. These AI chatbots leverage on machine learning techniques to study vast amounts of text from articles, books and webpages to identify patterns and structures of human language – allowing it to have wide-ranging applications including content generation, explaining complex concepts, and even taking and passing medical exams [9, 10].

Given the widespread adoption of ChatGPT, it is foreseeable and inevitable that a significant proportion of patients may independently seek answers to their medical queries from ChatGPT due to its accessibility and ability to provide personalized responses [11]. At the same time, some clinicians have also highlighted ChatGPT’s potential as a tool to enhance patient education due to its vast knowledge-base and ability to generate coherent and original responses [12, 13]. Despite this, there remain legitimate questions and concerns regarding the accuracy and reliability of responses generated by ChatGPT, as some have observed that the chatbot may generate false and biased information or even conjure up non-existent sources in its responses [14]. Furthermore, ChatGPT does not “reason” or “think” in a similar way to humans, instead generating responses based on recognized patterns and structures within the text it was trained with [15]. As such, it is also important to evaluate the relevance of ChatGPT’s responses – responses generated should be targeted and effective in answering the question at hand, rather than providing an excess of irrelevant information, which may overwhelm the patient.

Thus, our study aims to evaluate the accuracy and relevance of ChatGPT’s responses to FAQs regarding TKR to assess its clinical utility as a tool for patient education and preoperative decision-making. Our hypothesis is that ChatGPT will be able to provide factually accurate and relevant responses to these FAQs.

## Methods

### Frequently asked questions (FAQ)

A list of 50 clinically relevant FAQs regarding TKR was curated after discussion with three consultant orthopaedic surgeons (WC, GL, and MT) and with reference from commonly asked questions regarding TKR on Google web search. Google web search is one of the most used search engines worldwide and it utilizes AI algorithms to recognize patterns in user queries, allowing Google to predict and suggest commonly associated queries regarding a topic [5, 16, 17]. The search term “total knee replacement” was entered into Google web search on a newly installed browser to generate frequently associated questions under the “People also ask” box.

These FAQs were then classified into the following categories: (1) general/procedure-related, (2) indications for surgery and outcomes, (3) risks and complications of surgery, (4) pain and post-operative recovery, (5) specific activities after surgery and (6) alternatives and TKR variations (such as partial knee replacement, robotic TKR and bilateral TKR).

### Evaluation of ChatGPT responses

Each FAQ was individually input as prompts to ChatGPT (version 3.5) accessed on an internet browser, with the first response generated for each prompt recorded. Next, two consultant orthopaedic surgeons (GL and MT) independently rated each response based on its factual accuracy as well as the relevance of the response (Table 1). Factual accuracy was defined as the degree to which the response was scientifically true and up to date as of June 2023, and it was graded using a Likert scale from 1 to 5 (1 – very inaccurate, 2 – inaccurate, 3 – somewhat accurate, 4 – accurate, 5 – very accurate). Relevance was defined as the degree to which the response was helpful and effective in answering the question and was similarly graded using a Likert scale from 1 to 5 (1 – very irrelevant, 2 – irrelevant, 3 – somewhat relevant, 4 – relevant, 5 – very relevant). In the event of significant disagreement between the two raters (defined as a difference of two or more grades on the Likert scale), a third consultant orthopaedic surgeon (WC) was involved to review the response and adjudicate to award a final grade.

**Table 1:** Likert scale for grading factual accuracy and relevance, as well as their categorical classification

Factual accuracy		Relevance	
Likert grade	Accuracy (categorical)	Likert grade	Relevance (categorical)
1 – Very inaccurate	Inaccurate	1 – Very irrelevant	Irrelevant
2 – Inaccurate		2 – Irrelevant	
3 – Somewhat accurate		3 – Somewhat relevant	
4 – Accurate	Accurate	4 – Relevant	Relevant
5 – Very accurate		5 – Very relevant	

### Statistical analysis

Next, the ordinally rated responses were dichotomized using a threshold on the Likert scale (Table 1). For factual accuracy, responses were classified as accurate if they received an average or final grade of 4 or greater, whereas the rest of responses were classified as inaccurate. Similarly, for relevance, responses were defined as relevant if they received an average or final grade of 4 or greater, whereas the rest of responses were classified as irrelevant. Data analysis was performed using R software version 4.0.3 (R Foundation for Statistical Computing, Vienna, Austria, 2019). Inter-rater reliability was measured using Gwet’s AC2, as it has been shown to be a stable metric that is not significantly influenced by the distribution or prevalence of outcomes [18, 19].

## Results

### Overall performance

ChatGPT performed well overall, achieving a mean Likert grade of 4.6/5 for factual accuracy and 4.9/5 for relevance across all 50 questions. Overall, 44/50 (88%) of responses were classified as accurate and 50/50 (100%) of responses were classified as relevant. There was good inter-rater reliability as measured by Gwet’s AC2, with coefficients of 0.85 for factual accuracy and 0.94 for relevance. Three responses had significant disagreement (defined as ≥ 2 on the Likert scale) between the two raters which required the involvement of a third rater.

### General and procedure-related information

There were 9 FAQs relating to general and procedure-related queries for TKR (Table 2). Of the responses, 7/9 (77.8%) were classified as accurate (mean grade 4.5), and 9/9 (100%) were classified as relevant (mean grade 4.9). Responses to two procedure-related questions: “Do I need to fast before a total knee replacement?” and “Will I be awake during a total knee replacement?” were assessed to be inaccurate, with an average Likert grade of 3.5 and 3, respectively.

**Table 2:** General and procedure-related FAQs

	Factual accuracy		Relevance	
Question	Mean grade	Accurate^1^	Mean grade	Relevant^2^
What is a total knee replacement?	5	Y	5	Y
What can I do to prepare for a total knee replacement?	5	Y	5	Y
What is the implant material used in a total knee replacement?	5	Y	5	Y
How long is the scar from a total knee replacement?	5	Y	5	Y
Do I need to fast before a total knee replacement?	3.5	N	4.5	Y
How long does a total knee replacement surgery take?	5	Y	5	Y
What happens in a total knee replacement surgery?	5	Y	5	Y
Will I be awake during a total knee replacement?*	3*	N	5	Y
What types of anaesthesia can be used during a total knee replacement?*	4*	Y	5	Y
	4.5/5	7/9	4.9/5	9/9

*Denotes responses where there was significant disagreement (≥ 2 on the Likert scale) between the two reviewers and the final grade was awarded by a third reviewer

^1^Categorical outcome for accuracy, whereby accurate responses are defined as those with a mean or final grade of ≥ 4

^2^Categorical outcome for relevance, whereby accurate responses are defined as those with a mean or final grade of ≥ 4

### Indications for surgery and outcomes

There were 7 FAQs regarding the indications for TKR and the outcomes from surgery (Table 3). These questions relate to the indications for TKR and addresses its benefits and postoperative outcomes. Of the responses, 7/7 (100%) were classified as accurate (mean grade 4.9), and 7/7 (100%) were classified as relevant (mean grade 4.9).

**Table 3:** FAQs about TKR indications and outcomes

	Factual accuracy		Relevance	
Question	Mean grade	Accurate^1^	Mean grade	Relevant^2^
When is a total knee replacement necessary?	4.5	Y	5	Y
Am I a candidate for total knee replacement?	5	Y	4.5	Y
What is the ideal age to have a total knee replacement?	5	Y	5	Y
Can you be too young or too old to have a total knee replacement?	5	Y	5	Y
What are the benefits of a total knee replacement?	5	Y	5	Y
What is the success rate of a total knee replacement?	5	Y	5	Y
How long does a total knee replacement last?	4.5	Y	5	Y
	4.9/5	7/7	4.9/5	7/7

^1^Categorical outcome for accuracy, whereby accurate responses are defined as those with a mean or final grade of ≥ 4

^2^Categorical outcome for relevance, whereby accurate responses are defined as those with a mean or final grade of ≥ 4

### Risks and complications

There were 4 FAQs regarding the risks and complications from TKR (Table 4). Of the responses provided by ChatGPT, 4/4 (100%) were deemed to be accurate (mean grade 4.9), and 4/4 (100%) were deemed to be relevant (mean grade 4.6).

**Table 4:** FAQs about risks of TKR

	Factual accuracy		Relevance	
Question	Mean grade	Accurate^1^	Mean grade	Relevant^2^
Is total knee replacement a safe operation?	5	Y	5	Y
What are the risks of a total knee replacement?	4.5	Y	4*	Y
What is the risk of severe complications or death from a total knee replacement?	5	Y	5	Y
What medical conditions increase the risk of a total knee replacement?	5	Y	4.5	Y
	4.9/5	4/4	4.6/5	4/4

*Denotes responses where there was significant disagreement (≥ 2 on the Likert scale) between the two reviewers and the final grade was awarded by a third reviewer

^1^Categorical outcome for accuracy, whereby accurate responses are defined as those with a mean or final grade of ≥ 4

^2^Categorical outcome for relevance, whereby accurate responses are defined as those with a mean or final grade of ≥ 4

### Pain and post-operative recovery

There were 13 FAQs regarding pain during and after surgery and the post-operative recovery process (Table 5). These questions address perioperative pain and mitigation strategies, as well as the typical expected recovery process of a patient undergoing TKR. Of the responses, 12/13 (92.3%) were deemed to be accurate (mean grade 4.7), and 13/13 (100%) were deemed to be relevant (mean grade 5.0). The response to one question pertaining to postoperative recovery – “How much weight can I put on my operated leg after total knee replacement?” – was deemed to be inaccurate, with a mean Likert grade of 2.5.

**Table 5:** FAQs about pain and post-operative recovery after TKR

	Factual accuracy		Relevance	
Question	Mean grade	Accurate^1^	Mean grade	Relevant^2^
How painful is a total knee replacement?	5	Y	5	Y
Will I experience pain after total knee replacement?	5	Y	5	Y
How long will the pain and swelling last after a total knee replacement?	5	Y	5	Y
What types of painkillers will I get after a total knee replacement?	4.5	Y	5	Y
How much weight can I put on my operated leg after total knee replacement?	2.5	N	5	Y
When should I call my doctor or seek medical attention after total knee replacement?	5	Y	5	Y
How long do I need to stay in hospital after a total knee replacement?	5	Y	5	Y
How long will I need to be followed up with after a total knee replacement?	4.5	Y	5	Y
Are there any food restrictions after a total knee replacement?	4.5	Y	5	Y
What happens after total knee replacement?	5	Y	5	Y
How long does the recovery process take after a total knee replacement?	5	Y	5	Y
Will I need rehabilitation after a total knee replacement?	5	Y	5	Y
How long will the wound take to heal after a total knee replacement?	4.5	Y	5	Y
	4.7/5	12/13	5/5	13/13

^1^Categorical outcome for accuracy, whereby accurate responses are defined as those with a mean or final grade of ≥ 4

^2^Categorical outcome for relevance, whereby accurate responses are defined as those with a mean or final grade of ≥ 4

### Specific activities

There were 10 FAQs regarding the ability to perform specific activities such as walking, running and driving after TKR (Table 6). Of the responses, 10/10 (100%) were deemed to be accurate (mean grade 4.8), and 10/10 (100%) were deemed to be relevant (mean grade 5.0).

**Table 6:** FAQs about specific activities after TKR

	Factual accuracy		Relevance	
Question	Mean grade	Accurate^1^	Mean grade	Relevant^2^
When will I be able to walk after a total knee replacement?	5	Y	5	Y
When can I bathe and shower after a total knee replacement?	5	Y	5	Y
Are there any activity restrictions after a total knee replacement?	4.5	Y	5	Y
Can I run after a total knee replacement?	5	Y	5	Y
Can I sit cross-legged after a total knee replacement?	4	Y	5	Y
When can I drive after a total knee replacement?	5	Y	5	Y
When can I return to normal activities are a total knee replacement?	5	Y	5	Y
When can I resume playing sports after a total knee replacement?	4.5	Y	5	Y
When can I travel by plane after a total knee replacement?	5	Y	5	Y
Will I be able to squat or kneel after a total knee replacement?	5	Y	4	Y
	4.8/5	10/10	5/5	10/10

^1^Categorical outcome for accuracy, whereby accurate responses are defined as those with a mean or final grade of ≥ 4

^2^Categorical outcome for relevance, whereby accurate responses are defined as those with a mean or final grade of ≥ 4

### Alternatives/others

There were 7 FAQs regarding alternatives to TKR and variants of TKR such as bilateral TKR, robotic TKR and partial knee replacement (Table 7). Of the responses, 4/7 (57.1%) were deemed to be accurate (mean grade 4.1), and 7/7 (100%) were deemed to be relevant (mean grade 4.6). Responses deemed to be inaccurate include questions such as “Are there any alternatives to a total knee replacement?”, “What is robotic total knee replacement?” and “What is the benefit of robotic knee replacement?”, with all three questions having a mean Likert grade of 3.5.

**Table 7:** FAQs regarding alternatives and variations of TKR

	Factual accuracy		Relevance	
Question	Mean grade	Accurate^1^	Mean grade	Relevant^2^
What is the difference between a total knee replacement and a partial knee replacement?	5	Y	4.5	Y
Are there any alternatives to a total knee replacement?	3.5	N	4.5	Y
What is robotic total knee replacement?	3.5	N	4.5	Y
What is the benefit of robotic knee replacement?	3.5	N	4	Y
Is there any difference in recovery between conventional and robotic total knee replacement?	4.5	Y	4.5	Y
What are the advantages and disadvantages of bilateral total knee replacement?	4.5	Y	5	Y
I have osteoarthritis in both knees; should I consider doing total knee replacements for both knees at the same time?	4.5	Y	5	Y
	4.1/5	4/7	4.6/5	7/7

^1^Categorical outcome for accuracy, whereby accurate responses are defined as those with a mean or final grade of ≥ 4

^2^Categorical outcome for relevance, whereby accurate responses are defined as those with a mean or final grade of ≥ 4

## Discussion

Our results have shown that ChatGPT performed well overall in providing accurate and relevant responses to FAQs regarding TKR. Of the responses, 44/50 (88.0%) received a rating of “accurate” and “very accurate” from all assessors, indicating that ChatGPT was able to provide scientifically accurate responses for most FAQs. ChatGPT also performed well in providing relevant and helpful answers, with 50/50 (100%) of responses received a rating of “relevant” and “very relevant” from all assessors. Our findings are supported by existing studies which have demonstrated ChatGPT’s effectiveness in other specialties – Samaan et al. found that ChatGPT provided comprehensive responses to 86.8% of questions regarding bariatric surgery, while Deiana et al. found that ChatGPT was able to provide accurate responses to questions regarding vaccination myths and misconceptions 85.4% of the time [20, 21]. To our knowledge, our study is the first to critically evaluate ChatGPT responses for FAQs regarding TKR.

Despite its promise, our results also highlight that ChatGPT is not infallible – in our study, 6/50 (12.0%) of responses were found to be inaccurate (inaccurate responses highlighted in Additional file 1: Table S1). Indeed, several other studies have also highlighted a tendency for ChatGPT to sometimes provide inaccurate or misleading information, and at times even generate plausible-sounding falsehoods in a phenomenon coined “artificial hallucination” [14, 22, 23]. It is also important to highlight that ChatGPT is not capable of independent scientific reasoning and is only able to generate responses based on recognized patterns and structures in text it was trained with [15]. Lastly, another major criticism is that ChatGPT is only trained with available data up to September 2021 and thus may not be able to provide updated, real-time information to users [12, 23]. While many of these drawbacks are inherent to the available training data and the technology itself, continuous advancements in AI technology will mean that the accuracy and reliability of such chatbots will gradually improve. GPT-4, the latest iteration of ChatGPT, which was recently released in March 2023, has been shown to have significantly better performance, increased accuracy and superior reasoning skills compared with its past versions [24–26]. The introduction of plugins to GPT-4, which are additional functionalities from third-party applications, may also increase the utility and reliability of ChatGPT, allowing it to access up-to-date information from trusted sources such as peer-reviewed journals [27]. However, we chose not to use GPT-4 in our current study, as currently GPT-4 is only available with a paid subscription and thus is not freely available to the general public. As such, we used GPT-3.5, as we wanted our study to be reflective of what most patients will be able to use on a daily basis.

Despite its potential drawbacks, there are areas where ChatGPT can contribute and even excel at. Being an AI chatbot that is adaptive and readily accessible, ChatGPT is well suited in providing personalized information and medical advice to patients [28, 29]. Currently, ChatGPT supports more than 50 different languages and is able to adapt its responses based on factors such as the user’s age, education level and occupation (i.e. patients versus doctors) [30]. Furthermore, some studies have also shown that patients may in fact prefer ChatGPT responses to those given by human clinicians – rating its responses as significantly more empathetic [11]. Although direct supervision by a human clinician is still needed due to ChatGPT’s potential for mistakes, incorporation of this technology can greatly enhance and speed up the process of addressing patient queries and educating them about their medical conditions. Another area where ChatGPT can excel is in the generation of patient education materials. As a large language model trained on vast amounts of text, ChatGPT can easily generate coherent and original material in a matter of seconds [12, 31]. Lyu et al. demonstrated the ability of ChatGPT to translate radiology reports into plain language, while Mondal et al. showed that ChatGPT could write articles to educate patients on dermatological conditions [32, 33]. The involvement of ChatGPT in such processes, which are normally performed by human clinicians, can result in significant cost savings and improved efficiency for healthcare institutions.

There are several limitations in our study. First, we assessed ChatGPT’s responses using a curated list of 50 FAQs regarding TKR. This list of questions is not meant to be exhaustive, but rather as a proof-of-concept using the most frequently asked and clinically relevant questions. Furthermore, there might be slight differences between our list of FAQs and FAQs encountered in other countries due to variations in the prevalence and importance of different questions across different cultures and geographical regions. For example, questions about squatting or kneeling after TKR surgery might be more common in our local Singaporean population (a multi-ethnic Southeast Asian country) compared with Caucasian countries as such movements are part and parcel of daily life for many patients here [34]. Next, our study assessed the ability of ChatGPT to respond to FAQs about TKR to the average patient without providing additional patient-specific information. As such, we were not able to assess the ability of ChatGPT to provide personalized information and recommendations – an important aspect of clinical consultation and surgical counselling. In instances where patient-specific FAQs were asked (examples shown in Additional file 1: Table S2), we noted that ChatGPT was able to highlight its limitations and direct patients to speak to a doctor for a more detailed and personalized consultation. Follow-up studies should investigate the ability of ChatGPT and other AI chatbots in providing patient-specific and personalized information, and potentially even compare it to those provided by human clinicians. Lastly, while there are several other AI chatbots such as Google Bard and Microsoft Bing which may provide similarly informative responses with real-time data, our study chose to evaluate responses from ChatGPT, as it is currently the most popular and widely used AI chatbot on the market [35, 36]. Future studies should critically evaluate and compare the performances between these chatbots.

## Conclusion

ChatGPT performed well in providing accurate and relevant responses to FAQs regarding TKR, demonstrating great potential as a tool for patient education and preoperative decision-making. However, it is not infallible and can occasionally provide inaccurate medical information. Patients and clinicians intending to utilize this technology should be mindful of its limitations and ensure adequate supervision and verification of information provided.

### Supplementary Information

**Additional file 1: Table S1.** ChatGPT responses classified as inaccurate

**Additional file 2: Table S2.** Examples of ChatGPT responses to patient-specific FAQs

## Data Availability

Available on reasonable request.

## Declarations

### Declarations

Ethics approval and consent to participate.

Not applicable – no patient data was used.
