# Supplementary material for: Evaluating the accuracy and relevance of ChatGPT responses to frequently asked questions regarding total knee replacement
Source: Knee Surg Relat Res. 2024 Apr 2;36:15. doi: 10.1186/s43019-024-00218-5 (PMC10986046; doi:10.1186/s43019-024-00218-5)
Supplement: Supplementary file 1 — Additional file 1: Table S1. ChatGPT responses classified as inaccurate [file 43019_2024_218_MOESM1_ESM.docx]

Supplementary Table 1: ChatGPT answers classified as inaccurate (receiving a Likert grade of 3 or less for accuracy from at least 1 assessor)

| Question | ChatGPT answer | Remarks |
| --- | --- | --- |
| Are there any alternatives to a total knee replacement? | Yes, there are several alternatives to total knee replacement surgery. Depending on the cause and severity of your knee pain, your doctor may recommend one or more of the following alternatives:  Non-surgical treatments: Non-surgical treatments such as physical therapy, exercise, weight loss, and medications (including over-the-counter pain relievers and corticosteroid injections) may help alleviate knee pain and improve joint function.  Arthroscopic surgery: Arthroscopic surgery is a minimally invasive surgical procedure that can be used to diagnose and treat knee problems such as torn cartilage or damaged ligaments.  Partial knee replacement: If only one part of your knee is damaged, a partial knee replacement may be an option. This surgery replaces only the damaged part of the knee joint with an artificial implant, leaving the rest of the joint intact.  Osteotomy: In some cases, an osteotomy may be recommended. This surgery involves reshaping the bone to redistribute weight across the knee joint, which can help relieve pain and delay the need for knee replacement surgery.  Cartilage restoration: If the knee pain is caused by damage to the cartilage, cartilage restoration procedures, such as microfracture or autologous chondrocyte implantation, may be recommended to stimulate the growth of new cartilage.  It's important to discuss all treatment options with your doctor to determine the best course of action for your individual needs and goals. | Alternatives to total knee replacement depend on the individual patient and their indications for surgery. Arthroscopic surgery and cartilage restoration have not been proven to be reliable alternatives in the management of severe osteoarthritis. |
| Do I need to fast before a total knee replacement? | Whether or not you need to fast before a total knee replacement surgery may depend on the specific instructions provided by your surgeon or anesthesiologist, as well as the hospital or surgery center where the procedure will take place. It is important to follow the specific fasting instructions provided by your healthcare team to ensure a safe and successful surgery.  In general, it is common for patients undergoing surgery to be instructed to avoid eating or drinking anything for several hours before the procedure. This is because eating or drinking anything, including water, can increase the risk of aspiration, a serious complication that can occur if stomach contents enter the lungs during anesthesia.  The specific fasting instructions may vary depending on factors such as the type of anesthesia being used, the patient's age and medical history, and the time of day the surgery is scheduled. Your surgeon or anesthesiologist will provide you with detailed instructions on when to stop eating and drinking before the surgery, and what medications you should take or avoid prior to the surgery.  It is important to follow these instructions closely to ensure a safe and successful surgery, and to minimize the risk of complications. If you have any questions or concerns about fasting before your total knee replacement surgery, be sure to discuss them with your healthcare team. | Regardless of whether general anaesthesia or spinal anaesthesia is administered, the recommendation would be for patients to fast before their surgery. This is because spinal anaesthesia may be ineffective, necessitating conversion to general anaesthesia. |
| Will I be awake during a total knee replacement? | It is unlikely that you will be awake during a total knee replacement surgery. Total knee replacement surgery is typically performed under either general anesthesia or regional anesthesia.  General anesthesia involves medication that will put you into a deep sleep, and you will not be conscious during the procedure. Regional anesthesia, such as a spinal or epidural anesthesia, can also be used to numb the lower half of the body while you remain awake. In some cases, the surgeon may also administer a mild sedative to help you relax during the procedure.  The decision about which type of anesthesia to use will depend on several factors, including your overall health, preferences, and the surgeon's recommendation. Your healthcare team will discuss the different options with you and help you make the best choice for your individual situation.  It is important to follow the specific instructions provided by your healthcare team regarding fasting, medication use, and other preparations leading up to the surgery to ensure a safe and successful procedure. If you have any concerns or questions about the anesthesia or the procedure, be sure to discuss them with your healthcare team. | The answer is not fully accurate, as it is likely that the patient will be awake during the surgery if spinal anaesthesia is administered. |
| How much weight can I put on my operated leg after total knee replacement? | After a total knee replacement, the amount of weight you can put on your operated leg will depend on a few factors, including your overall health, the extent of the surgery, and your surgeon's specific instructions. Generally, your surgeon will give you specific weight-bearing instructions and limitations based on your individual case.  In most cases, patients are initially allowed to put only a limited amount of weight on their operated leg, typically between 50% to 75% of their body weight. This is usually done with the aid of crutches, a walker, or other mobility devices to help take some of the weight off the knee joint and allow it to heal properly.  Over time, as your knee strength and mobility improve, your surgeon may gradually increase the amount of weight you can put on your operated leg. This can vary, but typically ranges from 75% to 100% of your body weight, and may take several weeks or months to achieve.  It is important to follow your surgeon's instructions carefully and avoid overloading your knee joint or pushing yourself too hard during the recovery period. Doing so can increase the risk of complications and slow down your recovery. If you have any questions or concerns about weight-bearing after your surgery, be sure to discuss them with your healthcare team. | Patients are generally allowed full weightbearing as tolerated after a routine total knee replacement. |
| What is robotic total knee replacement? | Robotic total knee replacement, also known as robotic-assisted total knee arthroplasty, is a surgical procedure that utilizes robotic technology to assist in performing a knee replacement surgery. It combines the skills of a trained orthopedic surgeon with the precision and guidance of a robotic system.  During a robotic total knee replacement, the surgeon begins by creating a 3D model of the patient's knee joint using advanced imaging techniques, such as CT scans or MRI. This digital model provides detailed information about the patient's anatomy and allows the surgeon to plan the surgery more accurately.  The robotic system consists of a robotic arm attached to the surgical instruments. The surgeon uses this system to guide and control the movements of the instruments during the procedure. The robotic arm is equipped with sensors and cameras that provide real-time feedback to the surgeon, assisting in precise implant placement and alignment.  The robotic system helps improve the accuracy and precision of various steps involved in knee replacement surgery, such as bone preparation, implant positioning, and soft tissue balancing. It allows the surgeon to make real-time adjustments based on the patient's specific anatomy, optimizing the alignment and fit of the artificial knee components.  Benefits of robotic total knee replacement may include:  Improved accuracy: The robotic system enables precise implant positioning, potentially leading to better overall alignment and function of the knee joint.  Customization: The surgeon can tailor the procedure to the patient's unique anatomy, optimizing the fit and function of the knee implant.  Reduced complications: The enhanced precision and control provided by the robotic system may help reduce the risk of complications, such as implant misalignment or instability.  Faster recovery: Some studies suggest that robotic-assisted knee replacement may result in shorter hospital stays and faster rehabilitation compared to traditional techniques.  It's important to note that robotic total knee replacement is a surgical technique and should be performed by a skilled orthopedic surgeon with expertise in robotic-assisted procedures. The specific robotic systems used can vary among hospitals and surgeons, with popular options including the MAKOplasty and NAVIO systems. | In the second paragraph, it is mentioned that robotic systems use advanced imaging techniques such as CT or MRI scans. However, currently only one major commercial system (Mako by Stryker) uses preop CT scans. The rest of the major commercially available robotic systems do not use preoperative advanced imaging. |
| What is the benefit of robotic knee replacement? | Robotic knee replacement offers several benefits compared to traditional knee replacement techniques. Here are some of the key advantages:  Enhanced Precision: Robotic systems provide a higher level of accuracy and precision during knee replacement surgery. The robotic arm and associated software allow for meticulous planning and execution of the procedure, resulting in improved alignment and positioning of the artificial knee components. This precision can lead to better long-term outcomes and increased longevity of the implant.  Customized Surgical Approach: With robotic knee replacement, surgeons can create a personalized surgical plan based on the patient's unique anatomy. Preoperative imaging is used to generate a 3D model of the patient's knee joint, enabling the surgeon to accurately assess the joint's condition, make precise measurements, and determine the optimal size and placement of the implants. This customization helps ensure a better fit and alignment of the artificial knee, potentially leading to improved function and range of motion.  Real-Time Feedback and Assistance: Robotic systems provide real-time feedback to the surgeon during the procedure. The robotic arm is equipped with sensors and cameras that continuously monitor and analyze the patient's anatomy, allowing the surgeon to make immediate adjustments as needed. This feedback loop helps maintain accuracy and facilitates precise bone cuts, implant positioning, and soft tissue balancing.  Reduced Complications and Revision Rates: The improved accuracy and precision of robotic knee replacement may lead to a reduction in complications and the need for revision surgeries. Proper alignment and positioning of the implants can help minimize issues such as implant wear, instability, and premature failure, which can contribute to the need for additional surgeries in the future.  Faster Recovery and Rehabilitation: While individual recovery times may vary, some studies suggest that robotic-assisted knee replacement may lead to shorter hospital stays, reduced post-operative pain, and faster rehabilitation compared to traditional techniques. The enhanced precision of the robotic system may contribute to improved soft tissue balance and stability, allowing patients to resume normal activities more quickly.  It's important to note that the benefits of robotic knee replacement can vary depending on the specific system used, the surgeon's expertise, and the patient's individual circumstances. It is advisable to consult with an experienced orthopedic surgeon to determine if robotic knee replacement is a suitable option for your particular case. | As per above, not all robotic systems use preoperative imaging to generate a 3D model of the knee.  Furthermore, reduced complication rates and faster recovery after robotic total knee replacement remains controversial. |
