# Supplementary material for: Evaluating the accuracy and relevance of ChatGPT responses to frequently asked questions regarding total knee replacement
Source: Knee Surg Relat Res. 2024 Apr 2;36:15. doi: 10.1186/s43019-024-00218-5 (PMC10986046; doi:10.1186/s43019-024-00218-5)
Supplement: Supplementary file 2 — Additional file 2: Table S2. Examples of ChatGPT responses to patient-specific FAQs [file 43019_2024_218_MOESM2_ESM.docx]

Supplementary Table 2: Examples of ChatGPT’s responses to individualized FAQs

| Question | ChatGPT answer | Remarks |
| --- | --- | --- |
| Am I a candidate for total knee replacement? | As an AI language model, I cannot diagnose medical conditions or determine if you are a candidate for a total knee replacement. However, I can provide general information that may help you understand if you may be a candidate.  The decision to have a total knee replacement is typically made by a doctor after a thorough evaluation of your condition, symptoms, and medical history. The doctor will consider a range of factors, such as the severity of your knee pain, the extent of damage to your knee joint, your age, overall health, and lifestyle factors.  In general, a total knee replacement may be recommended if you have severe knee pain, stiffness, and loss of function that has not improved with nonsurgical treatments, such as medication, physical therapy, and lifestyle modifications. The severity of your symptoms, as well as the extent of damage to your knee joint, will help your doctor determine if a total knee replacement is the right option for you.  Your doctor may also consider other factors, such as your age and overall health, to determine if you are a good candidate for the surgery. For example, patients with certain medical conditions or risk factors may not be suitable for surgery or may require additional precautions.  It's important to discuss all available treatment options with your doctor and ask any questions you may have about the procedure, potential risks, and expected outcomes to make an informed decision about your care. | ChatGPT highlights that it unable to determine a patient’s candidacy for total knee replacement and explains that it can only provide general information on factors which influence this decision. It also recommends patients to consult with a doctor for more detailed discussion. |
| Is total knee replacement a safe operation? | Like any surgical procedure, a total knee replacement carries some risks. However, with proper preparation and care, the procedure is generally considered safe and effective for most patients. Here are some factors that can affect the safety of a total knee replacement:  Surgical experience: The safety of the procedure can depend on the experience and skill of the surgeon performing it. It's important to choose a surgeon who is experienced in performing total knee replacements and has a good track record of successful surgeries.  Overall health: Patients with certain medical conditions or risk factors may be at higher risk of complications during or after the surgery. Your doctor will evaluate your overall health and medical history to determine if a total knee replacement is safe for you.  Anesthesia: Anesthesia is used during the surgery to keep the patient comfortable and pain-free. While anesthesia is generally safe, there are some risks associated with it, particularly for patients with certain medical conditions. Your doctor will evaluate your health and discuss the potential risks and benefits of anesthesia with you.  Infection: Like any surgery, a total knee replacement carries a risk of infection. To reduce the risk of infection, your surgeon will take steps to ensure that the surgical area is as clean as possible and may prescribe antibiotics before and after the surgery.  Blood clots: Surgery can increase the risk of blood clots in the legs or lungs. To reduce this risk, patients may be prescribed blood-thinning medications or special compression stockings to wear after the surgery.  It's important to discuss any concerns you may have about the safety of the procedure with your doctor. They can provide detailed information about the potential risks and benefits of a total knee replacement and help you make an informed decision about your care. | ChatGPT highlights that the safety of the operation can depend on various patient, procedural and surgeon factors and recommends patients to have an personalised and detailed discussion with their surgeons. |
| How painful is a total knee replacement? | Total knee replacement surgery is a major surgical procedure, and it is normal to experience some pain and discomfort during the recovery period. However, the amount of pain experienced by each individual can vary depending on several factors, including their overall health, the extent of the surgery, and their pain tolerance.  During the surgery, you will be given anesthesia to ensure that you are comfortable and pain-free during the procedure. After the surgery, you will be given pain medication to help manage your pain. Your healthcare team will closely monitor your pain level and adjust your medication as needed to ensure that you are comfortable.  It is important to follow your healthcare team's instructions closely regarding medication use, activity restrictions, and physical therapy to aid in your recovery and manage any pain or discomfort. With proper care and rehabilitation, most people are able to manage their pain and regain full function of their knee joint within a few months after surgery. | ChatGPT astutely points out that pain can be influenced by many factors including the patient’s own pain tolerance. It also highlights the pain management strategies typically employed by the medical team during and after a total knee replacement. |
| How long do I need to stay in hospital after a total knee replacement? | The length of your hospital stay after a total knee replacement can vary depending on a variety of factors, including your overall health and the specific details of your surgery. In general, however, most patients will stay in the hospital for 2-4 days after a total knee replacement.  During your hospital stay, you will be closely monitored by your healthcare team to ensure that you are recovering well from the surgery and that there are no complications. You may receive pain medications, antibiotics, and other treatments as needed, and you will likely be asked to participate in physical therapy and other rehabilitation activities to help improve your strength and mobility.  Before you leave the hospital, your healthcare team will provide you with detailed instructions on how to care for your knee, manage pain, and perform exercises at home to help aid in your recovery. You will also be given information on what signs of potential complications to watch out for and when to contact your healthcare team if you have any concerns.  It is important to follow these instructions carefully and attend all follow-up appointments to help ensure the best possible outcome from your surgery. | ChatGPT highlights that the length of hospital stay can depend on multiple factors but provides a general estimate of the estimated duration for the average patient. |
| I have osteoarthritis in both knees, should I consider doing total knee replacements for both knees at the same time? | The decision to undergo bilateral total knee replacement for osteoarthritis in both knees at the same time is a complex one that should be carefully evaluated in consultation with your orthopedic surgeon. While bilateral knee replacement offers certain advantages, it also carries increased risks and challenges compared to unilateral knee replacement. Here are some factors to consider:  Severity of Symptoms: If the osteoarthritis symptoms in both knees significantly affect your quality of life and daily activities, bilateral knee replacement may provide comprehensive relief and a faster recovery compared to two separate surgeries. However, if the symptoms in one knee are more severe or limiting, your surgeon may recommend addressing that knee first before considering the second knee.  Overall Health and Fitness: Bilateral knee replacement is more demanding on your body compared to unilateral knee replacement. Your overall health, fitness level, and ability to tolerate a more extensive surgery and rehabilitation process should be evaluated. Pre-existing medical conditions, such as heart or lung problems, may increase the risks associated with bilateral knee replacement.  Risks and Complications: Bilateral total knee replacement has a higher risk of complications, including infection, blood loss, and venous thromboembolism, compared to a single knee replacement. It is important to thoroughly discuss these risks with your surgeon to understand the potential challenges and benefits specific to your case.  Rehabilitation and Support: Rehabilitation and physical therapy play a crucial role in the success of knee replacement surgery. Consider the support system available to you during the recovery period, as bilateral knee replacement may require more intensive rehabilitation efforts compared to a single knee replacement.  Ultimately, the decision to undergo bilateral total knee replacement should be based on a thorough assessment of your individual condition, functional limitations, and the risks and benefits involved. It's essential to have an open and detailed discussion with your orthopedic surgeon to understand the potential outcomes, weigh the pros and cons, and make an informed decision that aligns with your specific needs and circumstances. | ChatGPT rightly points out that the decision to undergo a bilateral instead of unilateral total knee replacement is a complex one and depends on a multitude of factors – with either option having their own pros and cons. It provides a general outline of some of the considerations and recommends patients to consult their surgeon for a personalized discussion on the ideal option for them. |
